# Antioxidant potential, total phenolic and total flavonoid contents of *Rhododendron anthopogonoides* and its protective effect on hypoxia-induced injury in PC12 cells

**DOI:** 10.1186/s12906-015-0820-3

**Published:** 2015-08-18

**Authors:** Linlin Jing, Huiping Ma, Pengcheng Fan, Rongmin Gao, Zhengping Jia

**Affiliations:** Department of Pharmacy, Lanzhou General Hospital, Lanzhou Command of CPLA, Lanzhou, 730050 People’s Republic of China

**Keywords:** *Rhododendron anthopogonoides*, Antioxidant, Hypoxia, Oxidative stress, PC12 cells

## Abstract

**Background:**

*Rhododendron anthopogonoides* Maxim, a kind of traditional Tibetan medicine, has been used to remove body heat, body detoxification, cough, asthma, stomachic and swelling, eliminate abundant phlegm and inflammatory for a long time. In the present study, the total phenols and total flavonoid contents as well as antioxidative properties of the crude extract and solvent fractions of *R. anthopogonoides* were determined using seven antioxidant assays. Additionally, the protective effect of the extracts on hypoxia-induced injury in PC12 cells was also investigated.

**Methods:**

The content of total flavonoid and total phenolic was determined by the aluminum colorimetric method and Folin-Ciocalteu assay, respectively. *In vitro* antioxidant study, the effect of the crude extract and solvent fractions on total antioxidant activity, reducing power, DPPH radical scavenging, ABTS radical scavenging, superoxide radical scavenging, hydroxyl radical scavenging and nitric oxide radical scavenging were examined. The correlation between the phenolic and flavonoid content of the extracts and their antioxidant properties also analyzed. Furthermore, the protective effect of extracts on hypoxia-induced damage on PC12 cells was investigated by cell viability, lactate dehydrogenase (LDH) release, malondialdehyde (MDA) production and the activities of antioxidant enzymes.

**Results:**

Our results showed that ethyl acetate and n-butanol fractions had higher content of phenolics and flavonoid compounds than other fractions. Except ABTS radical assay, n-butanol fraction exhibited the strongest antioxidant activity. While the hexane fraction showed the lowest antioxidant activity. Ethyl acetate also presented excellent antioxidant activity, which was just lower than n-butanol fraction. Significant correlation between the phenolic, flavonoid content of the extract and fractions with antioxidant assay excluding ABTS, OH scavenging assay was observed. Moreover, ethyl acetate and n-butanol fractions showed protective effect in PC12 cell under hypoxia condition, while crude extract and water fraction had no protective effect. In contrast, hexane fraction exhibited strong cytoprotective effect. Further study indicated that pretreatment of PC12 cells with ethyl acetate and n-butanol fractions, prior to hypoxia exposure, significantly increased the survival of cells and the activities of SOD, CAT, GSH-Px and T-AOC, as well as reduced the level of LDH and MDA. The gathered data demonstrated that ethyl acetate and n-butanol fractions were able to protect PC12 cells against hypoxia induced injury through direct free radical scavenging and modulation of endogenous antioxidant enzymes.

**Conclusion:**

These findings suggested that ethyl acetate and n-butanol fractions of *R. anthopogonoides* had significant antioxidant activity and could prevent PC12 cells against hypoxia-induced injury. So it might be regarded as an excellent source of antioxidants and had great potential to explore as therapeutic agent for preventing hypoxia related sickness in future.

## Background

Oxygen (O_2_) is the key substrate in metabolism and essential for aerobic organisms to produce energy. Aerobic organisms deprived of adequate supply of oxygen can result in hypoxia [1]. Hypoxia can be a consequence in some pathological conditions, such as cardiovascular and cerebral ischemia, inflammation, pulmonary disorders and cancer, as well as in many physiological conditions including high altitude and physical exercise [2]. Therefore, eliminating the damage caused by hypoxia plays a critical role in prophylaxis and therapy these diseases.

Although the mechanism of injury induced by hypoxia is complicated, oxidative stress may be one of the causative factors. Under hypoxia condition, the production of reactive oxygen species (ROS) will increase [3, 4]. Overproduced ROS will attack DNA, lipids, and proteins, which leads to cell injury and apoptosis [5]. So the pharmacologic strategies targeted at scavenging ROS may be an effective way of eliminating the physiological damage induced by hypoxia.

Antioxidant is widely recognized as effective free radical scavenger and can eliminate the ROS by scavenging initiating radicals, breaking chain reaction, and binding of metal ions [6, 7]. It is reported that antioxidant could be used to treat some hypoxia related diseases [8–11]. Recently, much more attentions have been paid on natural antioxidants [12–14], especially flavonoids, and phenolic compounds originated from plants due to synthetic antioxidants have potential toxicological effects [7, 15, 16].

In China, especially in Tibet, plants have always been and still remain a vital source of therapeutics for various illnesses, especially oxidative stress related disorders [14]. Plants growing at high altitude are always in the state of oxidative stress condition, it is reasonable to believe that these plants possess exceptional features or compounds which help them to grow there. Therefore, it is wise to find compounds with excellent antioxidant and anti-hypoxia activity from endemic plants grown in Qinghai-Tibetan Plateau.

*Rhododendron anthopogonoides* Maxim, belonging to the family Ericaceae, is an endemic species of the Qinghai-Tibetan Plateau, where it grows on the damp sides of mountains at high altitude [17, 18]. Its aerial part has been used as traditional Tibetan medicine for removing body heat, body detoxification, cough, asthma, stomachic and swelling, eliminating abundant phlegm, and inflammatory [19, 20]. Chemical analysis discloses that crude drug contains lots of compounds with a majority represented by flavonoids, diterpenoids and essential oil [21–24]. Modern pharmacology studies shows that essential oil of *R. anthopogonoides* exhibits insecticidal and anti-bacterial activities [19]. To the best of our knowledge, the biological and pharmacological properties of other compounds in the herb have not been investigated.

The objectives of this study were to determine the total phenols content, total flavonoid contents and antioxidative properties of the crude and fractionated extracts of *R. anthopogonoides* by DPPH, ABTS, superoxide, hydroxyl and nitric oxide radical scavenging assay as well as reduce power and phosphomlybdenum assay. Additionally, the protective effect of the extracts on hypoxia-induced injury in PC12 cells was determined. This study also aimed to correlate the phenolic and flavonoid contents of the crude and fractionated extracts with their antioxidant properties.

## Methods

### Sample collection

The aerial part of *R. anthopogonoides* was purchased from Xining Sanjiangbao Business Co., Ltd (Xining, Qinghai) and identified by Prof. Yongjian Yang, School of Pharmacy, Lanzhou University. The voucher specimen (2010–136) has been deposited at the herbaria of the Department of Pharmacognosy.

### Reagents and chemicals

2,2-diphenyl-1-picrylhydrazyl (DPPH), 2,2′-azino-bis-(3-ethylbenzothiozoline-6-sulfonic acid) disodiumsalt (ABTS), nitro blue tetrazolium (NBT), phenazine methosulphate (PMS), nicotinamide adenine dinucleotide (NADH), 1-(4,5-dimethylthiazol-2-yl)-3,5-diphenylformazan (MTT), thiobarbituric acid (TBA) and trichloroacetic acid (TCA) were purchased from Sigma Chemical Co. Sodium nitroprusside, sulfanilamide, naphthylethylene diamine hydrochloride, ammonium molybdate, potassium persulfate (K_2_S_2_O_8_) were obtained from Aladdin Industrial Inc. All other chemicals and solvents were of analytical grade from commercial suppliers in China.

The measurement kits for lactate dehydrogenase (LDH), malondialdelyde (MDA), superoxide dismutase (SOD), catalase (CAT), glutathione peroxidase (GSH-Px) and total antioxidative capacity (T-AOC) were obtained from Nanjing Jiancheng Bioengineering Institute (Nan-jing, China). The BCA (bicinchoninic acid) protein assay kit was obtained from Thermo (Rockford, USA).

### Cell line and culture medium

PC12 cells (rat adrenal pheochromocytoma cells) originated from Experimental Animal Center of Fudan University (Shanghai, China). Cells were cultured in DMEM/F12 medium supplemented with 10 % fetal bovine serum, 100 units/ml penicillin and 80 mg/ml gentamicin at 37 °C in a 5 % CO_2_ incubator.

### Extraction and fractionation

Air-dried of *R. anthopogonoides* (200 g) was extracted with EtOH–H_2_O (2 L, 70:30, v/v) for 6 h at room temperature with constant stirring. This process repeated 3 times. The resulting extract was combined, filtered and concentrated in a rotary evaporator to generate crude extract (39.86 g, 19.93 % based on the weight of dried sample weight). The crude extract (35 g) was suspended in H_2_O (500 mL) and extracted successively with hexane, ethyl acetate and n-butanol. The extract from each agent was then filtered, concentrated under vacuum to generate hexane (3.51 g, 1.76 %), ethyl acetate (17.47 g, 8.74 %), n-butanol (7.93 g, 3.96 %) and the final aqueous fractions (6.29 g, 3.14 %).

### Determination of total phenolic and flavonoid content

Total phenolic content of extract was measured using a modified Folin-Ciocalteu procedure [25]. In brief, 100 *μ*l of various concentrations of extract were mixed with 1.0 ml of Folin–Ciocalteu reagent (previously diluted 10-fold with distilled water). After 5 min, 1.0 ml of 7.5 % sodium bicarbonate solution was added to the mixture and allowed to stand for 90 min at room temperature in the dark. The absorbance of the mixture was measured at 725 nm. A calibration curve was prepared using a standard solution of gallic acid and the total phenolic content was expressed as mg gallic acid equivalents pergram of extract (mg GAE/ g extract).

The flavonoid content was determined according to method of Hatamnia with a minor modification [26]. Briefly, 50 *μ*l of sodium nitrate solution (5 %) was added to 500 *μ*l of the extracts and allowed to react for 5 min. Then 50 *μ*l of 10 % aluminum chloride solution was added. Finally, 250 *μ*l of 4 % sodium hydroxide solution was added into the mixture 5 min later. The absorbance of the mixture was immediately recorded at 518 nm. A calibration curve was prepared using a standard solution of rutin and the total flavonoid content was expressed as mg of rutin equivalents pergram of extract (mg RU/ g extract).

### DPPH radical scavenging assay

The DPPH radical scavenging activity was carried out in a 96-well microplate using an Spectramax i3 Reader according to the Vaz’ method with some modifications [27]. 150 *μ*l of various concentrations of extract were added to 150 *μ*l of 0.1 mM DPPH radical solution in ethanol and incubated for 30 min in the dark at room temperature. The absorbance of mixed solution was measured at 517 nm using a microplate reader (Spectramax i3, Molecular Devices). Ascorbic acid (V_C_) was used as a positive control. DPPH radical scavenging activity was calculated using the equation below:

1 $$ \mathrm{DPPH}\ \mathrm{scavenging}\ \mathrm{effect}\ \left(\%\right) = \left[\left({\mathrm{A}}_1\hbox{-} {\mathrm{A}}_0\right)/{\mathrm{A}}_1\right] \times 100 $$

Where A_1_ was the absorbance of control (DPPH solution without sample) at 517; A_0_ was the absorbance at 517 of sample at different concentrations with DPPH. The antioxidant activity was expressed as EC_50_ (mg/ml), the concentrations of the sample required to cause a 50 % decrease of the absorbance at 517 nm. A lower EC_50_ value corresponded to a higher antioxidant activity.

### ABTS radical scavenging assay

The ABTS assay was based on the method of Re with some modifications [28]. ABTS^+•^ stock solution was prepared by reacting equal volumes of 7 mM ABTS solution with 2.45 mM potassium persulfate solution. Then the mixture was place in the dark for 16 h at room temperature to yield a dark-colored solution. Before use, the stock solution was diluted with methanol to give an absorbance of 0.70 ± 0.02 at 734 nm. The test samples (100 *μ*l) at different concentrations were added to 3.9 ml of ABTS^•+^ working solution. After incubated for 10 min at room temperature in the dark, the absorbance of resulting solution at 734 nm was measured. Inhibition of ABTS radical was calculated using the eq. (1).

### Hydroxyl radical scavenging assay

The hydroxyl radical scavenging activity of extracts was determined according to the method of Halliwell with some modifications [29]. Test sample was prepared in distilled water. Reaction solution contained 100 *μ*l of deoxyribose (28 mM) in phosphate buffer (50 mM, pH 7.4), 100 *μ*l of EDTA (1 mM), 100 *μ*l of FeCl_3_ (1 mM), 100 *μ*l of H_2_O_2_ (1 mM) and 1 ml of sample solution with various concentrations. Then 100 *μ*l of ascorbic acid was added to initiate the reaction. After incubation at 37 °C for 1 h in water bath, the reaction was stopped by adding 500 *μ*l of 10 % trichloroacetic acid (TCA). The pink chromogen was developed by addition 500 *μ*l of 0.5 % thiobarbituric acid (TBA) in NaOH aqueous solution (50 mM) and heated in boiling water bath for 30 min. After cooling to room temperature, the absorbance of the solution was recorded at 532 nm. Ascorbic acid was used as a positive control. The capacity of extract on scavenging the hydroxyl radical was calculated according to the eq. (1).

### Superoxide radical scavenging assay

The superoxide scavenging activity was determined by PMS-NADH-NBT system with slightly modifications [30]. 50 *μ*l of nitro blue tetrazolium (NBT) solution (0.2 mM in distilled water), 50 *μ*l of NADH solution (0.5 mmol/L in 0.1 M Tris-HCl, pH 8.0) and 100 *μ*l of extract with different concentrations were mixed and treated with 50 *μ*l of phenazine methosulphate (PMS) solution (25 *μ*M PMS in distilled water). The reaction mixture was incubated at room temperature for 10 min, and the absorbance at 570 nm was measured. Ascorbic acid was used as positive control. The percentage of scavenging was calculated by the eq. (1).

### Nitric oxide radical scavenging assay

The nitric oxide radical scavenging assay was measured by Griess reaction with some modifications [31]. In brief, 50 *μ*l of various concentrations of fractions was added to 50 *μ*l of sodium nitroprusside (10 mmol/l in phosphate buffer, PH = 7.4). The reaction mixture was incubated under light at room temperature for 150 min. After incubation, 50 *μ*l of 0.33 % (w/v) sulfanilamide (in 20 % glacial acetic acid) was added and kept for 10 min. Then 50 *μ*l 0.1 % (w/v) naphthylethylene diamine hydrochloride was added and the resulting solution was further incubated for 30 min. The absorbance was measured at 540 nm in a microplate reader. Ascorbic acid was used as reference standard. The nitric oxide radicals scavenging activity of fractions were calculated according to the eq. (1).

### Reduce power assay

The reduce power assay of the extract fractions was determined by method of Oyaizu [32]. 100 *μ*l of various concentrations of extract were mixed with 2.5 ml of sodium phosphate buffer (0.2 M, pH 6.6) and 2.5 ml of 1 % (w/v) potassium ferricyanide. The mixture was incubated for 30 min at 50 °C in a water bath follow by addition 2.5 ml of 10 % trichloroacetic. The mixture was centrifuged at 3000 rpm for 10 min. The upper layer fraction (2.5 ml) was mixed with 2.5 ml of distilled water and 0.5 mL of 0.1 % ferric chloride. The absorbance was read at 700 nm after 10 min. Ascorbic acid was used as a positive control. A higher absorbance indicated a higher reducing power. EC_50_ value (*μ*g/ml) was the effective concentration giving an absorbance of 0.5 for reducing power and was obtained from linear regression analysis.

### Phosphomlybdenum assay (total antioxidant activity)

Total antioxidant activity of the extract was determined by the phosphomolybdate method according to Prieto [33]. 100 *μ*l of various concentrations of extract were combined with 1 ml of reagent solution (0.6 M sulfuric acid, 28 mM sodium phosphate and 4 mM ammonium molybdate). The reaction mixture was incubated for 90 min at 95 °C in a water bath. Then the resulting solution cooled to room temperature quickly. The absorbance of resulting solution was measured at 695 nm. EC_50_ value (*μ*g/ml) was the effective concentration giving an absorbance of 0.5 for phosphomlybdenum assay and was obtained from linear regression analysis.

### MTT assay

The cells were seeded onto 96-well culture plates at 1.0 × 10^5^ cells/well and incubated at 37 °C for 24 h. The medium was removed and fresh DMEM/F12 containing the appropriate dilution of compound was added into well. All extracts were dissolved in DMSO and added to the medium at a final concentration of 50, 100, 200 *μ*g/ml, respectively. After 1 h of incubation, the cells were cultured for 24 h in hypoxia incubator (2 % O_2_, 5 % CO_2_ and 93 % N_2_). At the end of the hypoxia exposure, the cells were incubation with 0.5 mg/ml MTT, 200 *μ*l serum-free medium for 4 h. Finally, 100 *μ*l of DMSO was added. The absorbance at the test wavelength of 570 nm was measured using a microplate reader (Model 550, Bio-Rad Laboratories, Inc). Cell viability was reported as percentage of the non-hypoxia control considered as 100 %.

### LDH release measurement

The PC12 cells were seeded in 90-mm culture dish at a density of 1x10^5^/ml and incubated for 24 h. Cells were treated with extracts in the same way as described above. At the end of hypoxia exposure time, 100 *μ*l of the culture supernatants were collected to a well, and LDH activity was detected at 450 nm by the commercial assay kits (Jiancheng Institute of Biotechnology, Nanjing, China). The LDH activity was expressed as U/mL.

### Lipid peroxidation assay

After hypoxia treatment, the cells were washed and homogenized. The homogenate was centrifuged and the total protein content was determined with the BCA protein assay kit. MDA contents were measured according to the direction of the assay kit (Jiancheng Institute of Biotechnology, Nanjing, China). The MDA results were expressed as nmol/mg protein.

### Analyses for SOD, GSH-Px, CAT and T-AOC activity

SOD, GSH-Px, CAT and T-AOC activities were performed using the commercial assay kits (Jiancheng Institute of Biotechnology, Nanjing, China). Detailed operation process was performed according to the manufacturer’s instructions. The SOD, GSH-Px, CAT and T-AOC activity was expressed as U/mg protein.

### Statistical analysis

Results were expressed as mean ± standard deviation (SD). For *in vitro* antioxidant assays, One-way analysis of variance (ANOVA) and Tukey’s test were used for statistical comparison. For biochemical investigations assay, data were analyzed using ANOVA followed by Dunnett’s test. Difference was regarded as statistically significant when *p* < 0.05.

## Result and discussion

### Extract and fraction yields

70 % ethanol, which is the most preferred solvent to extract phenolic compounds from plants, was used to extract the components in the *R. anthopogonoides.* Table 1 showed the yield of crude extract and solvent fractions of *R. anthopogonoides*. The highest yield was found in the ethyl acetate fraction, while hexane fraction exhibited the lowest yield. The results indicated that moderately polar compounds in ethyl acetate fraction were the main components of *R. anthopogonoides*.

**Table 1:** Total phenolic, flavonoid and extraction yield of ethanolic extract and solvent fractions of *R. anthopogonoides*

Extract/fractions	Extract yield (%)	Total flavonoid (mg RU/ g extract)	Total phenlics (mg GAE/ g extract)
Crude extract	19.93 %	231.37 ± 4.56^c^	165.00 ± 19.39^c^
Hexane	1.76 %	35.07 ± 0.51^a^	62.00 ± 2.82^b^
Ethyl acetate	8.74 %	264.71 ± 19.10^d^	218.36 ± 25.84^d^
n-butanol	3.97 %	283.55 ± 12.92^d^	225.16 ± 25.77^d^
Water	3.15 %	48.60 ± 3.10^b^	39.26 ± 6.00^a^

Value represents as mean ± SD of triplicate experiments. Values in the same column followed by a different letter (a–d) are significantly different (*p* < 0.05)

### Amount of phenolic and flavonoid content in *R. anthopogonoides*

Phenolic and flavonoids are the most important plant secondary metabolites and wildly spread throughout the plant kingdom [34]. It is universally accepted that many biological activities and health benefits of plants may be attributed to the antioxidant activity of flavonoids and phenolic compounds they contained. Therefore, the total phenolic and flavonoids content in plant extracts were investigated. Table 1 showed the total phenolic and flavonoids content in the crude extract and fractions. The contents varied among different extracting solvents used. Ethyl acetate and n-butanol fractions had higher phenolic and flavonoids contents than hexane and water fractions. The result represented that extract using moderately polar solvent was more effective than low or high polarity solvents to get higher content of phenolic and flavonoids.

### Determination of DPPH radical scavenging activity

**Fig. 1:**
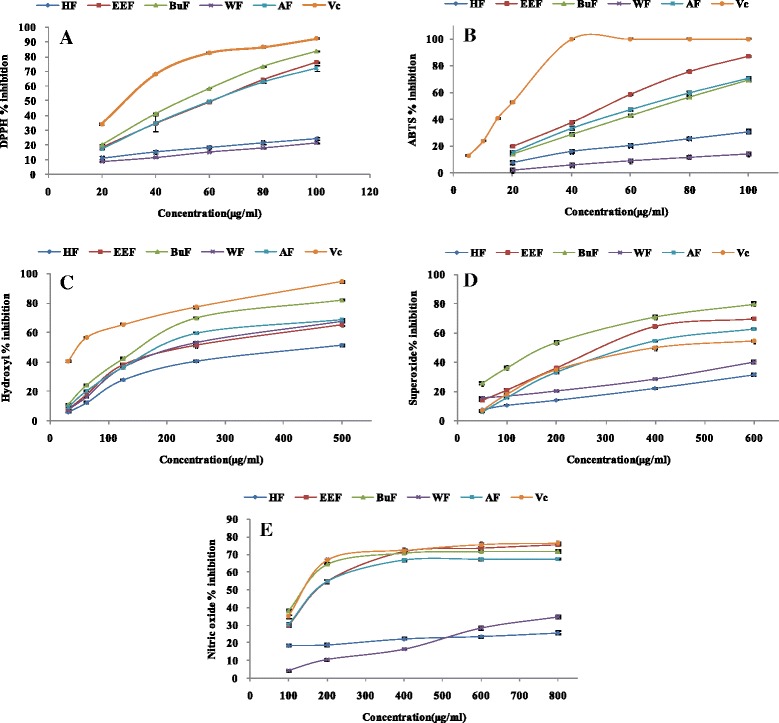
Antioxidant activities of crude extract and solvent fractions of *R. anthopogonoides*. Each value represents as mean ± SD of triplicate experiments. **a** DPPH radical scavenging effect, **b** ABTS radical scavenging effect, **c** The OH scavenging effect, **d** Nitric oxide radical scavenging effect, **e** Superoxide radical scavenging effect. CE: Crude extract, HF: n-hexane fraction, EEF: ethyl acetate fraction, BuF: n-butanol fraction, WE: water fraction, Vc: ascorbic acid

DPPH radical scavenging assay has been widely used to evaluate free radical scavenging activity of antioxidants as it is simple and high sensitive. DPPH is a nitrogen-centered radical and can accept electron or hydrogen radical from antioxidant to form a stable diamagnetic molecule. The color of DPPH solution will changed from purple to yellow which can be monitored as a decrease in absorbance at 517 nm [35]. As shown in Fig. 1a and Table 2, the scavenging effects of samples on DPPH radical were in the following order: n-butanol > ethyl acetate > crude extract > hexane > water. The highest scavenging effect was observed in n-butanol fraction with an EC_50_ of 53.11 ± 0.12 *μ*g/ml, but still weaker than ascorbic acid. The hexane and water fraction exhibited weaker DPPH radical scavenging activity with EC_50_ values were 259.65 ± 13.04 and 278.35 ± 1.4 *μ*g/ml, respectively. This study revealed that n-butanol and ethyl acetate fractions exhibited excellent DPPH radical scavenging activity.

**Table 2:** Effect of crude extract and solvent fractions of *R. anthopogonoides* on different free radical scavenging assays

Extract/fractions	EC_50_ value (*μ*g/ml) of radical scavenging				
	DPPH racidal	ABTS radical	Superoxide radical	Hydroxyl radical	Nitric oxide radical
Crude extract	63.73 ± 0.95^d^	69.96 ± 0.92^c^	483.00 ± 2.65^d^	204.65 ± 4.26^c^	197.38 ± 4.25^d^
Hexane	259.65 ± 13.04^e^	168.64 ± 2.50^d^	>1000	468.93 ± 16.17^f^	>1000
Ethyl acetate	61.78 ± 0.11^c^	53.36 ± 0.53^b^	433.33 ± 7.68^b^	240.39 ± 2.50^d^	183.83 ± 2.34^c^
n-butanol	53.11 ± 0.12^b^	71.32 ± 0.50^c^	270.67 ± 0.58^a^	145.87 ± 0.83^b^	151.34 ± 3.67^b^
water	278.35 ± 1.4^e^	345.22 ± 12.62^e^	>1000	232.95 ± 2.87^e^	>1000
Ascorbic acid	30.39 ± 0.23^a^	18.51 ± 0.13^a^	450.32 ± 6.28^c^	50.73 ± 1.83^a^	126.72 ± 7.24^a^

Each value represents as mean ± SD of triplicate experiments. Values in the same column followed by a different letter (a–f) are significantly different (*p* < 0.05)

**Table 3:** Correlations between the EC_50_ values of antioxidant assays and phenolic and flavonoids content of *R. anthopogonoides*

Assays	Correlation r	
	Flavonoids	Phenolic
EC_50_ of DPPH radical scavenging ability	−0.987^b^	−0.969^b^
EC_50_ of ABTS radical scavenging ability	−0.828	−0.876
EC_50_ of O_2_^−^ radical scavenging ability	−0.997^b^	−0.968^b^
EC_50_ of OH radical scavenging ability	−0.722	−0.611
EC_50_ of NO radical scavenging ability	−0.992^b^	−0.957^a^
EC_50_ of reduce power	−0.995^b^	−0.968^b^
EC_50_ of phosphomlybdenum assay	−0.868	−0.884^a^

r: correlation coefficient. ^a^indicates significance at *p* < 0.05, ^b^indicates significance at *p* < 0.01

### Determination of ABTS radical scavenging activity

The ABTS^•+^, generated by reacting ABTS with potassium persulphate, has been widely used to evaluate the antioxidant activity of hydrogen-donating antioxidants (scavenging aqueous phase radicals) and of chain breaking antioxidants (scavenging lipid peroxyl radicals) [36]. In the ABTS scavenging assay, all the fractions of *R. anthopogonoides* scavenged ABTS^•+^ in a concentration-dependent way (Fig. 1b). According to the EC_50_ value (Table 2), the order of ABTS radical scavenging ability was: ethyl acetate (53.36 ± 0.53 *μ*g/ml) > crude extract (69.96 ± 0.92 *μ*g/ml) > n-butanol (71.32 ± 0.50 *μ*g/ml) > hexane (168.64 ± 2.50 *μ*g/ml) > water (345.22 ± 12.62 *μ*g/ml). Although the ethyl acetate fraction showed the best ABTS radical scavenging activity, it still lower than Vc (18.51 ± 0.13 *μ*g/ml).

### Hydroxyl radical scavenging activity

Hydroxyl radical is regarded as the most reactive free radical among ROS and can nonspecifically damage almost all classes of bio-macromolecules in living cells. Many diseases, including atherosclerosis, diabetes, cancer, alzheimers disease and ageing, are contribute to the oxidative damage caused by hydroxyl radicals [37]. In the present study, Fe^3+^/Ascorbate/EDTA/H_2_O_2_ system was used to measure the hydroxyl radical scavenging activity of extracts. Hydroxyl radicals (OH), generated from Fenton’s reaction, attacked the deoxyribose to generate malondialdehyde (MDA), which could react with TBA to form a pink chromogen with maximum absorbance at 530 nm. Antioxidant could compete with deoxyribose to react with hydroxyl radicals, inhibit degradation of deoxyribose, reduce the formation of MDA and decrease the absorbance at 530 nm. Lower A_530nm_ value indicated the higher hydroxyl radical scavenging activity. Figure 1c described inhibition percentage of crude extract and fractions of *R. anthopogonoides*. The scavenging activity on hydroxyl of various solvent extracts decreased in the order of n-butanol > crude extract > ethyl acetate > water > hexane with significantly different EC_50_ values ranging from 145.87 ± 0.83 to 468.93 ± 16.17 *μ*g/ml, which were significantly higher (*p* < 0.05) than ascorbic acid (50.73 ± 1.83 *μ*g/ml). This result showed that all fractions displayed potential inhibitory effect of hydroxyl radical scavenging activity but lower than ascorbic acid.

### Superoxide radical scavenging activity

Superoxide radical, the product of one-electron reduction of oxygen, is less reactive than hydroxyl radicals and functions as a precursor of most ROS species and mediator in oxidative chain reactions. It was assumed that O_2_^·-^ could be the primary target of antioxidants against oxidative stress [38]. In this study, NADH-PMS-NBT system was used to determine the superoxide anion scavenging activities of extracts. In the PMS-NADH-NBT system, superoxide anion, generated from dissolved O_2_ by PMS-NADH coupling reaction, reduces NBT (yellow) to formazan (blue) which can be measured at 570 nm [39]. A decrease absorbance after addition of the antioxidant was a measure of its superoxide scavenging activity. As can be seen in Fig. 1d, the scavenging activity of all fractions was correlated well with the increase of concentrations. The n-butanol fraction exhibited the highest superoxide anion scavenging ability with EC_50_ value was 270.67 ± 0.58 *μ*g/ml, which was lower than ascorbic acid (IC_50_ = 450.32 ± 6.28 *μ*g/ml). This was followed by ethyl acetate fraction and crude extract. Hexane and water fractions only showed a very little superoxide anion scavenging ability.

### Nitric oxide radical scavenging activity

Nitric oxide (NO) is an essential bioregulatory molecule with many physiological functions, such as blood pressure regulation, neural signal transmission, control vasodilatation, smooth muscle relaxation and immune response. While excessive NO can interact with superoxide anion to form peroxynitriteion (ONOO^−^), which will inhibit mitochondrial respiratory chain enzymes, decrease cellular oxygen consumption, and prevent sodium transport across membranes [40]. The nitric oxide radical scavenging activity of extracts was measured by the greiss reaction. NO, generated spontaneously from aqueous solution of sodium nitroprusside at physiological pH, could interact with oxygen to produce nitrite ions which were monitored by griess reagent. Components with nitric oxide radical scavenging capacity could compete with oxygen to react with nitric oxide thereby inhibiting the generation of nitrite ions, so the absorbance at 550 nm would decrease. In this study (Fig. 1e), the NO scavenging capacities of samples and the positive control increased with the increase of concentration. At the highest concentration (800 *μ*g/ml), the percentage inhibition of n-butanol and ethyl acetate fractions were 75.51 and 71.81 % respectively, which were lower than ascorbic acid (76.23 %). The n-butanol fraction exhibited the highest NO scavenging ability with EC_50_ value was 151.34 ± 3.67 *μ*g/ml, following by ethyl acetate fraction and crude extract. The water and hexane fractions showed the lowest NO scavenging capacity.

### Reduce power assay

**Fig. 2:**
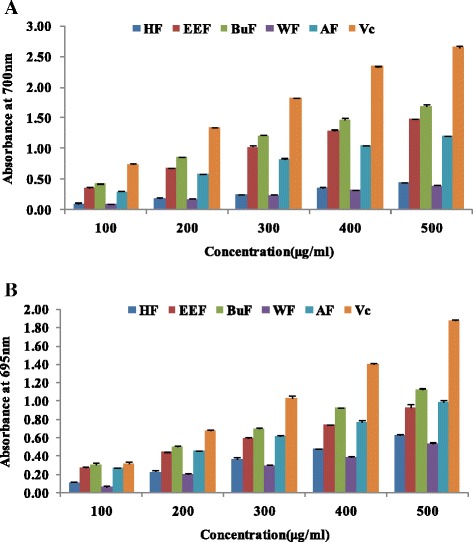
The reduce power (**a**) and phosphomlybdenum assay (**b**) of crude extract and solvent fractions of *R. anthopogonoides.* Each value represents as mean ± SD of triplicate experiments. CE: Crude extract, HF: n-hexane fraction, EEF: ethyl acetate fraction, BuF: n-butanol fraction, WE: water fraction, Vc: ascorbic acid

The reducing capacity of a sample may serve as a significant indicator of its potential antioxidant activity. The extracts with electron donation ability can reduce Fe^3+^ into Fe^2+^, which can be monitored by measuring the formation of Perl’s Prussian blue at 700 nm [41]. The increase in the absorbance indicated an increase in the reducing power activity. As shown in Fig. 2a, the reducing capacities of samples and the positive control increased with the increase of concentration. The n-butanol fraction exhibited the highest reducing activity, following by ethyl acetate fraction, while the lowest reducing activity was found in hexane and water fractions.

### Phosphomlybdenum assay (total antioxidant activity)

The total antioxidant capacity of the crude extracts and fractions was measured spectrophotometrically through phosphomolybdenum method, which is based on the reduction of Mo (IV) to Mo (V) by the sample and the subsequent formation of green phosphate/Mo (V) compounds with a maximum absorption at 695 nm under acidic pH conditions [42]. The antioxidant capacity of the crude extract and fractions of *R. anthopogonoides* was found to decrease in the order n-butanol > crude extract > ethyl acetate > hexane > water (Fig. 2b).

### Correlation between the contents of total phenolic, total flavonoid and antioxidant activity of *R. anthopogonoides*

Phenolic and flavonoid are always considered to be the major contributors for the antioxidant activity of plant materials [43]. The antioxidant activity of them was mainly due to their redox properties, which allow them to act as reducing agents, hydrogen atom donors, singlet oxygen scavengers and transition metal ions chelator [43]. Therefore, the correlation between the total phenolic, total flavonoid content and the antioxidant activity was determined using Pearson’s correlation test. As shown in Table 3, a significant correlation was found between the phenolic, flavonoid content of the extracts with the DPPH, O_2_^−^, NO radical-scavenging activity, reduce power and phosphomlybdenum assay. However, a non-significant correlation was observed between the ABTS, OH scavenging activity and the total phenolic and flavonoid content of these extracts. The reason of this result might be raised from the interference by the chemical structure of phenolic and flavonoids and the other chemical components in the extract. General speaking, ethyl acetate and n-butanol fractions with higher levels of phenolic, flavonoids content exhibited higher antioxidant ability. Meanwhile, hexane and water fractions contained the lowest phenolic and flavonoids contents and owned the lowest antioxidant value. These results indicated that the antioxidant activity of extracts of *R. anthopogonoides* may be related, at least in part, to the presence of high content of flavonoids compounds and other phenolics.

### MTT assay

**Fig. 3:**
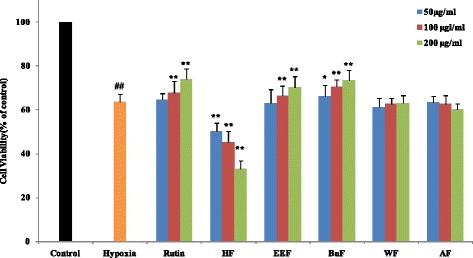
Effects of crude extract and solvent fractions of *R. anthopogonoides* on cell viability in hypoxia-injuried PC12 cell. PC12 cells are pre-incubated with EEF or BuF (50–200 *μ*g/ml) for 1 h prior to hypoxia for 24 h. After the treatment, cell viability is determined by MTT analysis. Data are shown as means ± SD (n = 6). ^##^ *p* < 0.01 vs. control.^*^ *p* < 0.05 vs. hypoxia, ^**^ *p* < 0.01 vs. hypoxia. CE: crude extract, HF: n-hexane fraction, EEF: ethyl acetate fraction, BuF: n-butanol fraction, WE: water fraction, Vc: ascorbic acid. Rutin is used as positive control

The cytotoxic and proliferative effects of crude extract and fractions on PC12 cells under hypoxia were investigated using MTT assay. As shown in Fig. 3, the MTT assay demonstrated that PC12 cells were cultured under hypoxic conditions for 24 h reduced cell viability to 63.54 % compared to untreated cells (*p* < 0.01). This result was in accordance with previous reported by Luo [44]. Pretreatment of AF and WF had no influence on the growth of PC12 cell under hypoxia condition, suggesting that AF and WF had not any protective effect. However, pretreatment of HF could significantly decrease the cell viability of PC12 cells, suggesting the HF exhibited strong cytoprotective effect on PC12 cell. In contrast, the proliferation of PC12 cells treated with EEF and BuF could increase in a concentration-dependent manner. These results suggested that EEF and BuF exhibited protective effect against hypoxia-induced injury. When pretreated of EEF or BuF at concentration of 500 *μ*g/mL, the cell viability increased by 70.23 and 73.52 % respectively compared with the control. Given this result, 500 *μ*g/mL of EEF and BuF were selected for following experiments.

### LDH release measurement

**Fig. 4:**
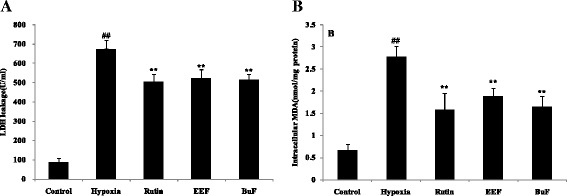
Effects of EEF and BuF on LDH release (**a**) and MDA content (**b**) in PC12 cells under hypoxia condition. PC12 cells are pre-incubated with 200 *μ*g/ml EEF or BuF for 1 h prior to hypoxia for 24 h. Data are shown as mean ± SD (n = 6). ^##^ *p* < 0.01 vs. control. ^**^ *p* < 0.01 vs. hypoxia. EEF: ethyl acetate fraction, BuF: n-butanol fraction. Rutin is used as positive control

LDH, which is a stable cytoplasmic enzyme present inside cells, will released into the cell culture supernatant when the cell membrane is damaged [45]. Therefore, LDH level in the supernatant could be used to access the extent of cellular damage and cytotoxicity. Whevbnn PC12 cell exposed to hypoxic for 24 h, there was a significant increase in LDH release compared to control cells (*p* < 0.01). However, incubation of cells with EEF and BuF remarkably blocked LDH leakage by 22.12 and 23.60 %, respectively, compared with the hypoxia group (Fig. 4a). The results demonstrated that EEF and BuF could maintain the integrity of cell membrane and inhibit the release of LDH from the PC12 cells to the culture supernatant.

### Lipid peroxidation assay

Lipid peroxidation is one of the most important mechanisms contributing to oxidative stress. MDA as a poisonous end product of free radical attacking on lipids is always regarded as an index of lipid peroxidation [46]. As shown in Fig. 4b, MDA was in low concentration under control group, while hypoxic for 24 h significantly increased MDA content by about 4 fold compared with control (*p* < 0.01). However, incubation of cells with EEF and BuF remarkably decrease MDA content by 32.01 and 40.29 %, compared with hypoxia group. The results showed that EEF and BuF could attenuate lipid peroxidation induced by hypoxia.

### Analyses for SOD, GSH-Px,CAT and T-AOC activity

**Fig. 5:**
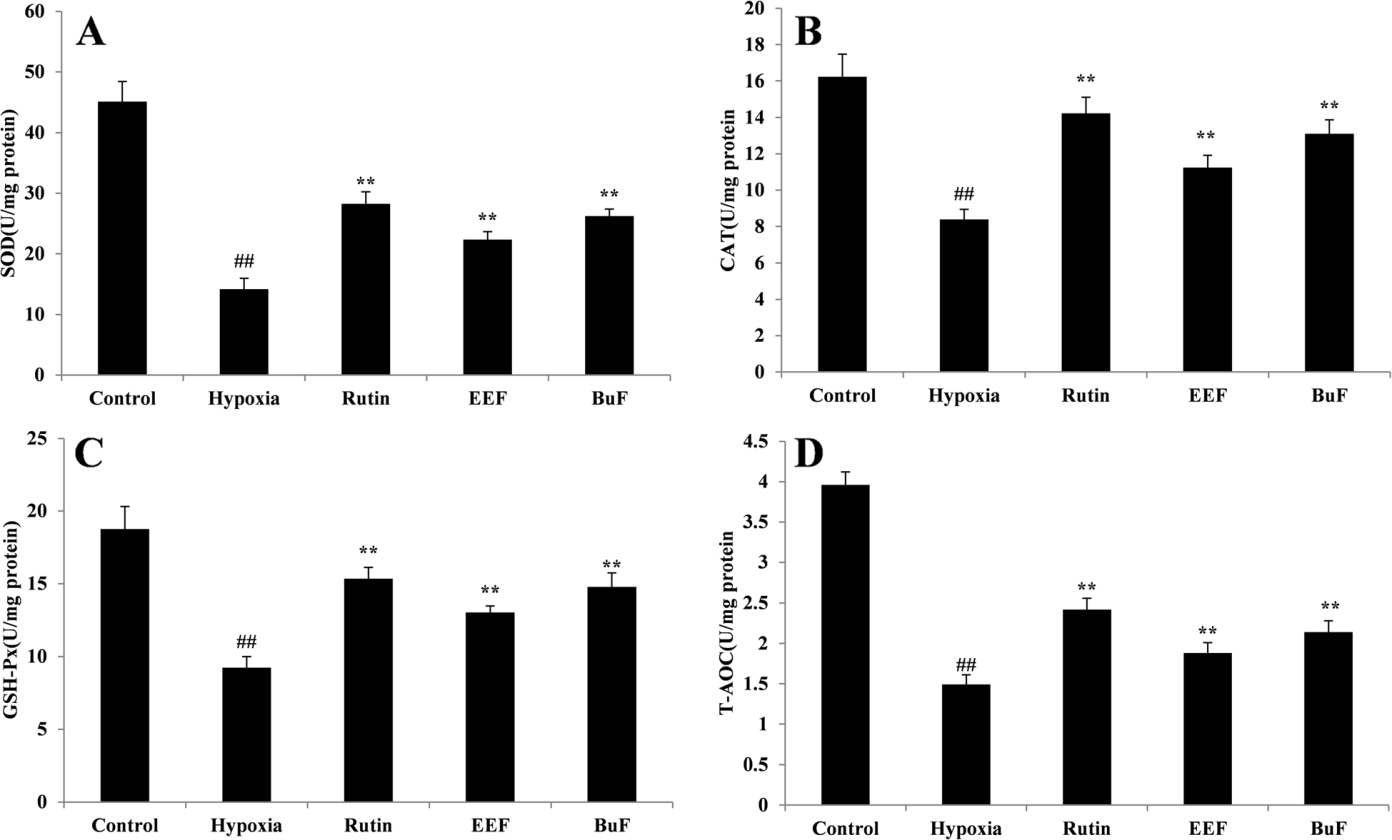
Effects of EEF and BuF on the activity of SOD (**a**), CAT (**b**), GSH-Px (**c**) and T-AOC (**d**) in PC12 cells under hypoxia condition. PC12 cells are pre-incubated with 500 *μ*g/L EEF or BuF for 1 h prior to hypoxia for 24 h. ^##^ *p* < 0.01 vs. control. ^**^ *p* < 0.01 vs. hypoxia. Data are shown as mean ± SD (*n* = 6). Rutin is used as positive control

It has been reported that hypoxia-induced cell death are partly mediated by oxidative stress. To mitigate cumulative burden of oxidative stress, cells generally utilize antioxidant defense systems to scavenge ROS [47]. SOD, GSH-Px and CAT are the first line of defense against oxidative stress and can inhibit free radical formation and prevent oxidative damage by ROS [15]. SOD is able to convert superoxide radical into H_2_O_2_, which can be decomposed to O_2_ by CAT and GSH-Px [48]. In this paper, to determine whether the protective effects of EEF and BuF on hypoxia-induced injury were mediated by their antioxidant functions, the activities of antioxidant enzymes in PC12 cell under hypoxia with or without pretreatment of EEF and BuF were investigated. As shown in Fig. 5, after exposure of PC12 cells under hypoxia for 24 h, the activities of SOD, CAT, GSH-Px and T-AOC significantly decreased by 68.51, 48.37, 50.75 and 62.37 %, respectively (*p* < 0.01 vs. control). Pretreatment of EEF and BuF, SOD, CAT, GSH-Px and T-AOC activity significantly increased compared with hypoxia group (*p* < 0.01). These results showed that pretreatment with EEF and BuF significantly attenuated the oxidative damage of PC12 under hypoxia, as reflected by maintaining the activity of antioxidant enzymes.

## Conclusions

In the present study, the antioxidant activity of *R. anthopogonoides* and its protective effect on hypoxia-induced oxidative damage in PC12 cells were reported for the first time. Our results showed that ethyl acetate and n-butanol fractions, which were rich in phenolics and flavonoid compounds, had stronger antioxidant capacity against seven antioxidant assays than other fractions and could be considered as a potential natural antioxidant. Moreover, ethyl acetate and n-butanol fractions showed protective effect on PC12 cells under hypoxia condition, while crude extract and water fraction did not shown any protective role. In contrast, hexane fraction exhibited strong cytoprotective effect. Further study indicated that ethyl acetate and n-butanol fractions could decrease LDH leakage and MDA level as well as restore the activity of SOD, CAT, GSH-Px and T-AOC in PC12 cells under hypoxia condition. The gathered data allowed concluding that ethyl acetate and n-butanol fractions were able to protect PC12 cells against the damage induced by hypoxia through direct free radical scavenging and modulation of endogenous antioxidant enzymes. Further work is in process to identify the bioactive constituents presented in the extract, which can be responsible for the antioxidant activity and anti-hypoxic effect of *R. anthopogonoides*.

## Abbreviations

ROS: Reactive oxygen species
DNA: Deoxyribonucleic acid
DPPH: 2,2-diphenyl-1-picrylhydrazyl
ABTS: 2,2′-azino-bis-(3-ethylbenzothiozoline-6-sulfonic acid) disodiumsalt
LDH: Lactate dehydrogenase
MDA: malondialdelyde
SOD: Superoxide dismutase
CAT: Catalase
GSH-Px: Glutathione peroxidase
T-AOC: Total antioxidative capacity
DMEM: Dulbecco’s Modified Eagle’s Medium
MTT: 3-(4,5-dimethylthiazol-2-yl)-2,5-diphenyl tetrazolium bromide
